# Mechanotransduction in Coronary Vein Graft Disease

**DOI:** 10.3389/fcvm.2018.00020

**Published:** 2018-03-14

**Authors:** Matthijs Steven Ruiter, Maurizio Pesce

**Affiliations:** ^1^Cardiovascular Tissue Engineering Unit, Centro Cardiologico Monzino (IRCCS), Milan, Italy

**Keywords:** vein graft disease, saphenous vein, CABG, mechanobiology, mechanotransduction, YAP, shear stress, strain

## Abstract

Autologous saphenous veins are the most commonly used conduits in revascularization of the ischemic heart by coronary artery bypass graft surgery, but are subject to vein graft failure. The current mini review aims to provide an overview of the role of mechanotransduction signalling underlying vein graft failure to further our understanding of the disease progression and to improve future clinical treatment. Firstly, limitation of damage during vein harvest and engraftment can improve outcome. In addition, cell cycle inhibition, stimulation of Nur77 and external grafting could form interesting therapeutic options. Moreover, the Hippo pathway, with the YAP/TAZ complex as the main effector, is emerging as an important node controlling conversion of mechanical signals into cellular responses. This includes endothelial cell inflammation, smooth muscle cell proliferation/migration, and monocyte attachment/infiltration. The combined effects of expression levels and nuclear/cytoplasmic translocation make YAP/TAZ interesting novel targets in the prevention and treatment of vein graft disease. Pharmacological, molecular and/or mechanical conditioning of saphenous vein segments between harvest and grafting may potentiate targeted and specific treatment to improve long-term outcome.

## Introduction

Autologous saphenous veins (SVs) are the most commonly used conduits in revascularization of the ischemic heart by coronary artery bypass graft surgery (CABG), due to their availability, accessibility for harvesting and easy handling when making anastomoses. Continuous improvements in surgical techniques have improved clinical outcome and short-term graft patency; on the other hand, the long term SV grafts have a cumulative 10 year patency of 60% due to graft disease (VGD), and are still outperformed by arterial grafts (1–6). In VGD, three subsequent phases are distinguished: thrombosis, which occurs typically within the first month, intima hyperplasia, which occurs up to 1 year after surgery, and finally atherosclerosis, which becomes predominant several years following CABG. SV graft patency is affected by many factors, ranging from surgical technique and SV preservation before implantation, to SV quality, patient comorbidities and medication, and is associated with quality of life (7–10). The role of mechanosensing in the progression of graft failure has been recognized and is predictive for later graft patency. However, the effects of individual factors remain hard to determine, due to the difficulty of addressing mechanical cues in experimental systems and *in vivo* (11, 12). In the circulation, blood vessels are exposed to several mechanical stimuli simultaneously, including shear stress on endothelial cells (ECs), luminal pressure and circumferential stretch, both of which exert their effect mainly on smooth muscle cells (SMCs), and longitudinal stretch. These conditions vary widely throughout the circulation, and regulate blood pressure, vascular permeability, and attraction and invasion of inflammatory cells. The interplay of all these factors determines the local vascular microenvironment, and dictates physiological and pathological remodelling of blood vessels. This is particularly relevant in VGD, when SVs are exposed to challenging conditions that are widely different from their native environment. Therefore, the current review aims to provide an overview of the role of the mechanobiology underlying VGD, with a focus on pathways participating in detection of physiologic and pathologic mechanical stress, in an attempt to further our understanding of the disease progression and to improve future clinical treatment.

## Vein Harvest and Engraftment

Both the surgical procedure and the new hemodynamic conditions have pervasive consequences for structure as well as function of the SV. Harvesting of the vein inevitably results in denervation and loss of external blood supply through *vasa vasorum*, inducing ischemia and potentially changing vasomotor tone and potentiating vasospasm (13). Subsequently, high-pressure distension is routinely performed to check for leakage and to reduce vasospasm. Together with ischemia and reperfusion injury after engraftment, this causes damage and loss of ECs and SMCs through oxidative stress and cytotoxic activation (14–17). The extent of damage is dependent on the type of harvesting; endoscopic techniques are associated with a lower rate of leg infection, whereas open vein harvesting (OVH) seems to be superior in terms of long-term patency. However, results from different studies have not been conclusive (18–21). The same limitations are true for traditional versus *no touch* harvesting, where the *no touch* technique seems better for graft patency by (i) leaving the peri-adventitial tissue, (ii) providing physical support, and (iii) maintaining the integrity of both endothelial and adventitial cells (4, 22, 23). Vessel damage during harvest and engraftment complicates the SV recovery and adaptation capacity to the new conditions, and the combination of SV excision and arterialization results in increased vasoconstriction and reduced endothelium-mediated vasodilation (24, 25).

## Adaptation to a New Hemodynamic Environment

Immediately after engraftment, SVs are challenged with hemodynamic conditions to which they need to adapt. Under native conditions, SVs experience low pressure loads (5–10 mmHg) and quasi-steady flow patterns with low shear stresses (0.1–0.6 Pa). By contrast, after CABG, SVs are subjected to high pulsatile pressure (120/80 mmHg) with a circumferential strain of 10–15%, a mean flow rate up to 250 ml/min, and a high wall shear stress (0.75–2.25 Pa) (26, 27). Opposite to arteries, SVs are anisotropic and become incompliant at high pressures (28). The increases in flow and shear stress together with wall tension, result in additional loss of ECs, damage to SMCs and extracellular matrix (ECM) alterations (29, 30). Stretching of SMCs disrupts actin bundles and results in a structure with scattered pores, followed by loss of SMC nuclei and actin filaments, inducing SMC proliferation (31). Subsequently, deposition of platelets and fibrin takes place, and leukocytes from the circulation infiltrate the vessel wall. Next, growth factors are released from platelets, SMCs and macrophages, which leads to SMC proliferation and migration to the intima, as well as ECM deposition, leading to intima hyperplasia (14, 29, 30).

## Untangling Mechanical Factors

The new hemodynamic environment exposes the SV to various different mechanical factors simultaneously, which can act either synergistically or antagonistically. Mechanical static deformations and stresses, increased pulsatile deformations and stresses, and altered shear stress, require the vein graft to acquire an artery-like structure with geometric remodelling and wall stiffening, but also induce intima hyperplasia and inflammation, which may induce failure in the long term (32).

Exposure to a combination of increased flow, pressure and shear stress leads to rearrangement of SMCs, both *ex vivo* and *in vivo* (31, 33). To untangle the separate effects of flow and pressure, several *ex vivo* perfusion models have addressed single or combined mechanical factors on SV adaptation and pathophysiology. Under continuous laminar flow, an increase in flow reduces intimal hyperplasia, while an increase in pressure induces intimal accumulation. When flow and pressure increase simultaneously, apoptosis becomes apparent in the vessel wall after day 1, resulting in lower cell density and media thinning. Some *ex vivo* models find intima accumulation after 1 week, but this is not completely consistent between models (33–39).

Exposure of SVs to arterial shear stress under low pressure induced expression and activity of matrix metalloproteinase (MMP)−2 and MMP-9, tissue inhibitor of metalloproteinase-1, as well as expression of plasminogen activator inhibitor-1 and osteopontin. Venous marker Ephrin B4 on the other hand, was attenuated under arterial shear stress (40, 41). Endothelial cells harbour a multitude of sensors on their apical, junctional, and basal surfaces to sense mechanical signals from the blood, which have key roles during developmental, physiological, and pathological processes. Mechanosensing molecules include junctional proteins, receptor kinases, integrins, focal adhesions, G-proteins and G-protein-coupled receptors, ion carriers, actin cytoskeleton, primary cilia, and the glycocalyx, enabling the vasculature to respond to changing demands within seconds or during the course of several days. The pathways through which the different sensors transduce the physical signals remains elusive, but integrins seem to play an important role. The mechanisms of shear stress sensing and transduction are reviewed extensively elsewhere (42–44). The effects of shear stress are mainly mediated by ECs. However, exposure of SMCs to shear stress cannot be excluded, due to increased pressure and fenestration, and in particular due to endothelial denudation early after engraftment (45). What is important to distinguish in the context of VGD, is the difference between the effects of laminar flow and disturbed flow on the ECs. In fact, laminar flow results in EC alignment, stress fiber formation, low proliferation, and elevated Krüppel-like Factor 2 expression, whereas NF-κB activation, expression of adhesion molecules ICAM-1 and VCAM-1, high EC turnover, and production of reactive oxygen species are the result of disturbed flow (43). SMCs are sensitive to deformations and strain induced by arterial pressure. Increases in pressure are associated with elevated vasoconstriction and attenuated nitric oxide-mediated dilatation (46). Veins perfused for 7 days under high pressure exhibited enhanced expression of transforming growth factor (TGF)-β1 and upregulation of microRNAs-138/200b/200 c, suppression of tissue inhibitor of metallo-protease-1, and equal or more intima hyperplasia than veins exposed to low pressure (40, 47). *In vitro* straining of isolated human cells has revealed that cyclic stretch of SMCs induces the number of cells and DNA synthesis, collagen, fibronectin and TGF-β expression, and MMP2 activity, while reducing αSMA and p27^kip1^ expression, resulting in a proliferative SMC phenotype (29, 48–50). These effects were detected in cells derived from SVs, but absent in cells from the internal thoracic artery (48, 49). Moreover, cellular responses are specific for the type of strain, indicated by Asanuma et al., who found that static but not cyclic stretch induced MMP-9 mRNA levels and MMP-2 mRNA, protein content and secretion (51). Interestingly, in rat SMCs, cyclic strain attenuated SMC proliferation and upregulated VEGF secretion, which could be beneficial for re-endothelialisation of SV grafts (52).

The role of adventitial cells in the development of VGD is acknowledged in animal models (53, 54), but remains elusive in the human pathology (55, 56). *Ex vivo*, CABG-like oxygen levels, i.e., high luminal oxygen levels, and deprivation of adventitial oxygen due to severing of the *vasa vasorum*, induced proliferation of adventitial microvasculature even before SMC proliferation, and resulted in neovascularization (57, 58). The adventitia of human SVs harbours CD34-positive cells, which display multilineage differentiation capacities, and were able to induce neovascularization in mice (59, 60). Study of the effects of mechanical factors on adventitial cell function is needed to elucidate the relevance of these cells in physiologic and pathologic remodelling in VGD. Key experimental findings in mechanotransduction relevant for VGD are summarized in Table 1.

**Table 1:** Key experimental findings of mechanotransduction relevant for VGD.

Year	Main finding	Reference
1989	Intima hyperplasia is associated with low flow velocity, whereas medial thickening is associated with increased circumferential strain *in vivo.*	(32)
1996	*Ex vivo* perfusion of SV segments induces proliferation and intima hyperplasia.	(61)
2000	Mechanical strain induces ECM protein synthesis and MMP activity *in vitro*.	(29)
2012	YAP/TAZ mediates SMC phenotypic modulation and neointima formation *in vitro* and *in vivo*	(62)
2013	Arterial shear stress in SVs *ex vivo* induces expression of MMPs, PAI-1 and osteopontin, attenuates ephrin B4. Increased pressure is associated with increased vasoconstriction and reduced dilatation.	(40)
2016	CABG-like oxygen conditions induce adventitial neovascularization in SVs *ex vivo*.	(58)
2016	YAP/TAZ mediates EC proliferation and inflammation induced by flow.	(63)

As in all vascular remodelling events, inflammatory mediators are involved in VGD. Both systemic and local inflammatory markers are associated with SV remodeling and VGD (64–69). Since these factors are, at the moment, only indirectly connected to mechanosensing, they fall beyond the scope of this review.

## YAP/TAZ Signalling

The Hippo pathway is a growth control signalling pathway first described in the context of developmental biology, but has since emerged as an important factor in uncontrolled cell growth in cancer. Yes-associated protein (YAP) and transcriptional co-activator with PDZ-binding motif (TAZ) are the main downstream effectors of the Hippo pathway regulating cell survival, proliferation and apoptosis. Activity of the YAP/TAZ complex is confined to cells exposed to stiffness of the surrounding extracellular matrix, localization at edges and curvatures, or cells undergoing mechanical straining (70). YAP and TAZ exert their effect by nuclear translocation and binding to transcriptional enhancer associate domain (TEAD), which induces YAP target gene transcription. Currently, evidence is accumulating that YAP and TAZ are involved in vascular remodelling and cardiovascular disease, such as pulmonary hypertension, atherosclerosis and restenosis (71). This may also implicate a role for YAP/TAZ in VGD.

In ECs, YAP is a transcriptional regulator that transduces signals from VE-cadherin mediated endothelial cell-cell contact to the nucleus. ECs exposed to disturbed flow, display YAP/TAZ activation and nuclear translocation with subsequent target genes upregulation, including ankyrin repeat domain 1 (ANKRD1), cysteine-rich angiogenic inducer 61 (CYR61), and connective tissue growth factor (CTGF), resulting in increased proliferation and decreased apoptosis of ECs. On the other hand, laminar flow suppressed YAP/TAZ, with downregulation of YAP target genes ANKRD1, CYR61, CTGF, baculoviral IAP repeat-containing 5, and angiopoietin-2. This suggests an atheroprotective role of the Hippo-kinase pathway that antagonizes the YAP/TAZ nuclear translocation by enhancing their phosphorylation levels (63, 72–75). In mice, EC-specific YAP/TAZ deletion led to a hyper-pruned vascular network, disrupted barrier integrity and reduced neovascularization (76). In accordance, YAP has been demonstrated to be critical in retinal angiogenesis (77, 78). Moreover, *in vivo* blockade of YAP/TAZ translation significantly suppressed endothelial inflammation and the size of atherosclerotic lesions in mice (79). YAP/TAZ inhibition in ECs suppressed c-Jun N-terminal kinase (JNK) signalling and downregulated expression of pro-inflammatory genes, reducing monocyte attachment and infiltration (79). In zebrafish, YAP nuclear translocation was shown to be transient, shuttling in or out of the nucleus within 10 min after starting or stopping flow. Interestingly, in this model, laminar flow induced nuclear translocation of YAP, even though this was during blood vessel formation (80).

In addition to shear-sensing ECs, YAP is implicated in SMC function, where YAP levels are consistently associated with proliferation, migration and a synthetic phenotype. In patient material, YAP was markedly lower in the aortic wall of patients with ascending aortic aneurysms compared with healthy aortic samples, while downregulation of YAP in SMCs was associated with ECM disorders of the media (81). In animal models of arterial injury, it was demonstrated that YAP levels are elevated in response to injury, correlating with a synthetic SMC phenotype (62, 82). In cultured rat cells, YAP knockdown impaired SMC proliferation and enhanced the expression of contractile SMC genes by upregulating myocardin expression (82). Overexpression of YAP induced SMC proliferation and migration, whereas genetic deletion of YAP attenuated the injury-induced phenotypic switch in SMCs and attenuated neointima formation in mice (62). In accordance, inhibition of YAP/TAZ function with RNA interference or Verteporfin significantly reduced vascular SMC proliferation (83). In breast cancer cell lines, the effect of YAP on proliferation is at least partially mediated by p27^kip1^, but this pathway has not yet been confirmed in vascular (mal)adaptation (84).

In addition to ECs and SMCs, the YAP/TAZ pathway is involved in the cellular response to stretch in mesenchymal stem cells (85). Evidence suggests that YAP can expand stem cell pools and induce reprogramming of differentiated cells to stem cell-like progenitor phenotypes (86). Finally, YAP is linked to platelet function, since platelet-released thromboxane A2 (TXA2) induces YAP activation, facilitating wound healing in response to vascular injury (87). Together, these studies indicate the broad role of YAP signalling in the mechanosensing of vascular tissue relevant in the development of VGD, and may provide an opportunity for therapeutic intervention. However, it is still to be unveiled how mechanical signals lead to changes in YAP and TAZ localization, in the cell cytoplasm under pathologic conditions (88–90). On the other hand, very recent findings suggest that force generation by docking stress fibres to focal adhesion contacts and translation of this tension to nuclear deformation and opening of the nuclear pores promote YAP nuclear translocation, thus connecting directly mechanosensing and YAP-dependent transcriptional circuitries (91, 92).

## Therapeutic Opportunities

As discussed elsewhere, the problem of SV graft patency remains a clinical priority (93, 94). A first improvement of clinical outcome may be obtained by limiting the initial damage to the SV. As discussed above, comparisons of open *vs.* endoscopic isolation, and traditional *vs.* no touch harvesting have not been conclusive, but are very relevant for future clinical practice. Open, no touch harvesting might be the least damaging technique, but this has to be confirmed by future research. However, a reduction of damage is not likely to prevent VGD completely. Therefore, additional therapy remains necessary with the clear primary outcome to increase the longevity of the vein grafts, and prevent re-interventions such as redo graft implantation or graft stenting, two risky procedures.

Since SMC hyperplasia is a hallmark of VGD, it seems worthwhile investigating the cell cycle. To support this notion, there are promising results from a single nucleotide polymorphism in the p27^Kip1^ gene. p27^Kip1^, together with other cyclin-dependent kinase inhibitors, is upregulated during vascular repair and negatively regulates growth of vascular SMCs *in vivo* (95, 96). The p27(kip1)−838AA genotype is associated with a reduction in coronary artery in-stent restenosis and improved patency of lower extremity bypass grafts, through inhibition of both SMCs and fibroblasts (55, 97, 98). Intervention in the cell cycle may be an interesting approach to inhibit SMC proliferation in VGD, and experience may be drawn from drug-eluting stent research (99).

**Figure 1:**
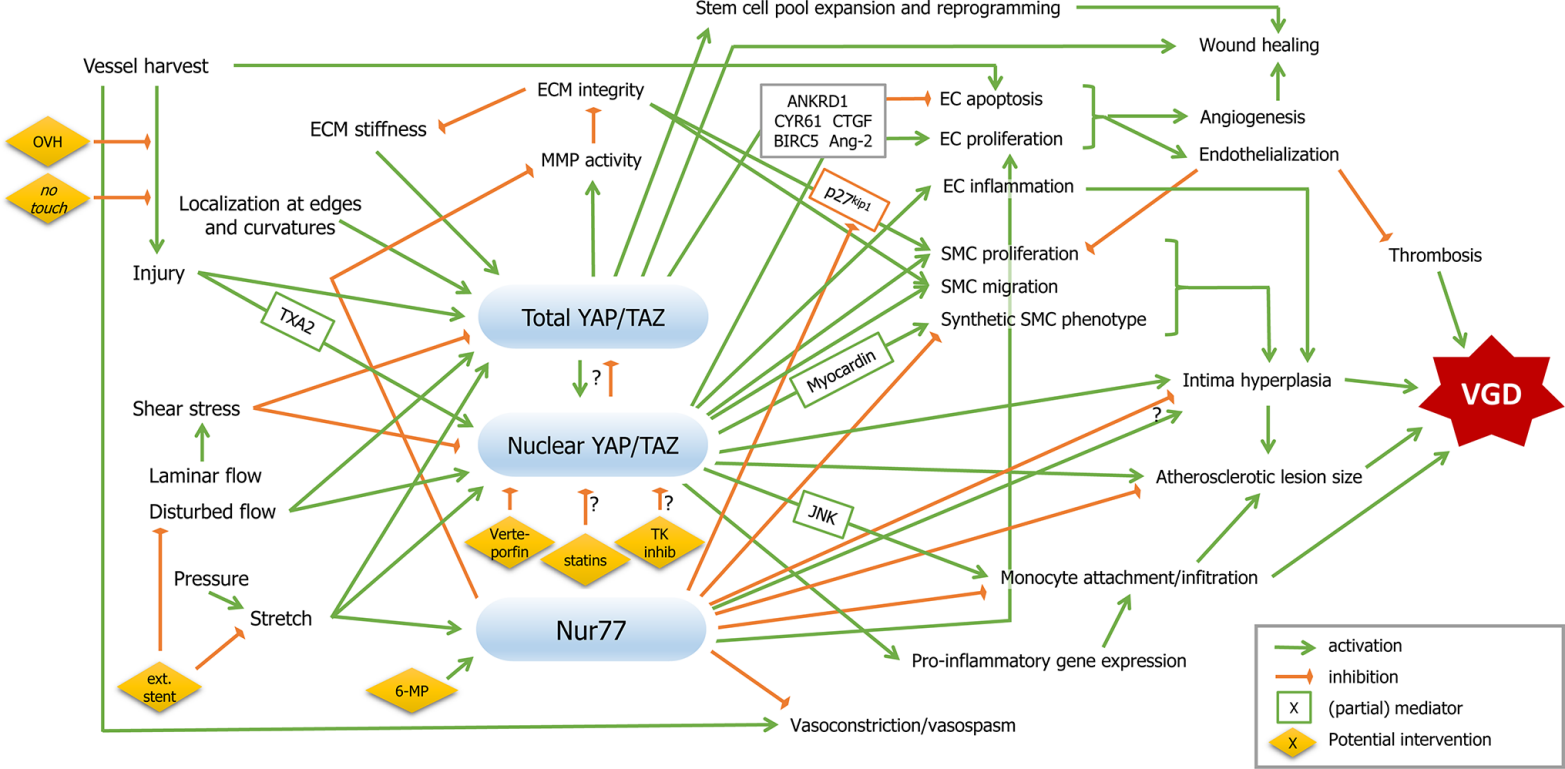
Mechanotransduction and therapeutic targets in coronary vein graft disease. Mechanical factors are depicted on the left, vein graft disease on the right, and signalling pathways described in this review in between. Signalling nodes are marked in blue. Green arrows indicate activation, orange inhibition. Boxed factors are (partial) mediators. Potential therapeutic interventions are presented in yellow diamonds. Abbreviations are explained in the main text.

Due to the complex nature of VGD, schematically represented in Figure 1, identification of therapies that target multiple cell types and/or are upstream from multiple processes, are most likely to lead to a robust inhibition of thrombosis, intima hyperplasia and/or atherosclerosis. Experimental evidence from vascular pathology, injury and adaptation suggests several options. Nuclear receptor Nur77 has demonstrated effects across multiple cell types in atherosclerosis, where it inhibits lesion formation by reducing SMC proliferation, reduces MMPs and attenuates macrophage accumulation and infiltration (100–103). In a rabbit model of in-stent restenosis, Nur77 activator 6-mercaptopurine (6-MP) inhibited intima hyperplasia, although this effect was not observed in a porcine model (104, 105). In addition, Nur77 enhances EC survival and lowers vasoconstrictor endothelin-1 (106, 107). Interestingly, Nur77 levels were elevated both by mechanical stretch of isolated venous SMCs, and by arterial pressure in vein segments, while activation of these receptors reduced SMC proliferation and elevated expression of contractile SMC markers (48). In contrast, in a rat carotid interposition model, Nur77 inhibition reduced intima hyperplasia (108). Therefore, more research is needed to establish the role and therapeutic potential of Nur77 in VGD.

An interesting approach, which might be beneficial in terms of both prevention and repair, is external stenting of SV grafts. Both *in vitro* and *ex vivo*, external stents consistently attenuated intima hyperplasia (109–112). In accordance, reduction of neointima formation was reported in a clinical study after 1 year, however without significantly affecting SVG failure rate (113). These responses are mediated by both strain and shear stress; clinically, oscillatory shear rate was lower in the stented group, and correlated with the development of diffuse intima hyperplasia, whereas *ex vivo* work implicated wall tension, which was lower in the stented group, attenuating intimal hyperplasia, medial SMC apoptosis and subsequent medial fibrosis (110, 114). Additional clinical results are needed to evaluate the efficacy of this promising treatment.

As discussed in the previous section, YAP levels and nuclear translocation play a central role in the translation of mechanical signals into cellular responses, not only in shear-sensing ECs and strain-sensitive SMCs, but also with a link to ECM components, stem cells and even the blood. Inhibition of YAP/TAZ signalling inhibits EC inflammation and SMC proliferation, and is associated with a contractile SMC phenotype and attenuation of atherosclerosis, which are all central elements in VGD. For therapeutic application of YAP inhibition, cancer research has identified several potent inhibitors, including Verteporfin, tyrosine kinase (TK) inhibitors and statins (115, 116). Simvastatin was associated with reduced neointima formation, which could partially be explained by inhibition of SMC proliferation and migration, and lowering of LDL levels and MMP-9 activity (117–119). In the CASCADE trial, statin therapy was associated with decreased intima accumulation after 1 year (120). The relation between statins and YAP however, was not investigated in these studies. Further research should shed light on the feasibility and efficacy of YAP inhibition in the prevention of VGD.

Regardless of the therapeutic target selected for prevention of VGD, CABG offers a unique opportunity for topical delivery of pharmacological, molecular or mechanical therapy. Between harvest and grafting, the SV segment is maintained in a physiological solution. Ideally, for the patient this time is kept as short as possible, but can still allow a window for treatment, allowing exposure to specific drugs or gene expression interfering strategies, e.g., siRNAs, antagoMIRs or agoMIRs (121). For example, agomiR-33 and adenovirus-mediated microRNA-21 gene transfer attenuated neointima formation in rat vein grafts (122, 123). Conversely, Smooth Muscle Enriched Long Noncoding RNA (SMILR) knockdown inhibited cell proliferation *in vitro* (124). As a different approach, more complex treatments can be considered, with a dedicated easy-to-use bioreactor, allowing mechanical preconditioning of the SV, exposure to different molecules/drugs on the luminal and adventitial side (58), or combinations thereof. For preconditioning or molecular/pharmacological pre-treatment, one could consider intervening in a mechanical event, in the cell cycle, in potential cell phenotypic modulation, in paracrine signalling, or in other processes. Depending on the putative target, the optimal delivery modality has to be devised. Pharmacological pretreatment seems a feasible approach and, from a technical point of view, is probably the least challenging method. It remains to be confirmed whether inhibition of YAP/TAZ signalling in this stage is beneficial. In this view, administration of Verteporfin, statins and TK inhibitors would provide a feasible and promising approach. In addition, oral statin treatment may be beneficial after CABG.

## Conclusions

Clinical therapy to prevent or attenuate the development of VGD are necessary to improve long-term SV patency. Refinements in harvesting and grafting techniques can limit the damage, but are not sufficient to prevent VGD. A better understanding of the mechanotransduction signalling pathways underlying this condition may give rise to the development novel therapeutic strategies. Due to the complex nature of VGD, upstream targets that affect multiple mechanisms are most likely to form a robust therapy. Inhibition of YAP/TAZ signalling, responsive to both shear stress and mechanical strain, reduces not only EC inflammation and SMC proliferation and migration, but also monocyte attachment and infiltration, and may therefore be an effective therapy for VGD. Pharmacological and/or mechanical conditioning of the SV between harvest and grafting should be considered, since it allows for targeted and specific treatment.

## Author Contributions

MR analyzed the literature and wrote the manuscript. MP wrote sections of the manuscript and critically reviewed the manuscript. Both authors contributed to manuscript conception and revision, and both read and approved the submitted version.

## Conflict of Interest Statement

The authors declare that the research was conducted in the absence of any commercial or financial relationships that could be construed as a potential conflict of interest.

The reviewer MF and handling Editor declared their shared affiliation.
